# Compressed Wideband Spectrum Sensing Based on Discrete Cosine Transform

**DOI:** 10.1155/2014/464895

**Published:** 2014-01-08

**Authors:** Yulin Wang, Gengxin Zhang

**Affiliations:** Institute of Communication Engineering, PLA University of Science and Technology, Yudao Street 14, Nanjing 210007, China

## Abstract

Discrete cosine transform (DCT) is a special type of transform which is widely used for compression of speech and image. However, its use for spectrum sensing has not yet received widespread attention. This paper aims to alleviate the sampling requirements of wideband spectrum sensing by utilizing the compressive sampling (CS) principle and exploiting the unique sparsity structure in the DCT domain. Compared with discrete Fourier transform (DFT), wideband communication signal has much sparser representation and easier implementation in DCT domain. Simulation result shows that the proposed DCT-CSS scheme outperforms the conventional DFT-CSS scheme in terms of MSE of reconstruction signal, detection probability, and computational complexity.

## 1. Introduction

In cognitive radio networks (CRNs), secondary CR users should fleetly and accurately sense the wideband spectrum, so that they can detect the unused spectrum holes, reconfigure their parameters to utilize the spectrum available, and avoid interference to primary users (PUs) [1, 2]. In practice, only a small part of the wideband spectrum is occupied by the PUs. Too high sampling rate required for scanning the wideband spectrum can cause immense computational costs and sensing problems. In compressed sampling (CS) [3], the sampling and compression operations are combined into a low complexity compressed sampling. For current CRNs, the CS has been used to alleviate the sampling burden, which aims at depressing the sampling rates for the acquisition of wideband signals [4].

To implement compressed wideband spectrum sensing, CRs need to exploit sparsity of signal in frequency domain. In the literature, [4] firstly applied CS for acquiring wideband signals using sub-Nyquist sampling rates, [5] exploited a structured compressed sensing, and [6] studied a power spectrum blind sampling (PSBS) algorithm trying to reconstruct the power spectrum. All of these systems belonged to the class of discrete Fourier transform (DFT) based compressed spectrum acquisition, which employed the complex exponential functions set as orthogonal sparse basis.

However, the DFT complex exponential matrix is not the only orthogonal basis that can be used to reconstruct wideband communication signals. The paper [7] proposed a cyclic spectrum based wideband spectrum estimating scheme considering the 2D sparse signal of interest in the cyclic spectrum domain. The paper [8] considered using an adaptive tree structured dictionary of orthogonal bases to optimize the compressive sensing recovery of image and audio signal. The paper [9] applied several time-frequency transforms including DFT, DCT, and discrete sine transform (DST) to spectrum sensing for cognitive power line communication (PLC) systems. However, there were few studies combining the CSS and DCT for simultaneous estimation of the spectrum occupancy states over a wide band.

A set of cosinusoidal functions can be used as an orthogonal sparse basis to implement the compressed spectrum sensing (CSS) scheme. Hence, we will synthesize the scheme as discrete cosine transform DCT-CSS and the conventional discrete Fourier transform (DFT) based CSS system as DFT-CSS in this paper. Although DFT-CSS algorithm could quite effectively reduce the sampling requirement, high computation complexity replaced in signal processing is a serious disadvantage. Furthermore, the CRNs require the sensing time to be short to ensure network responsiveness and efficiency. In this paper, our main contribution is to reconstruct a wideband spectrum signal from sub-Nyquist-rate compressive samples by DCT-CSS. The novel algorithm provides more accurate recovery and lower computation complexity.

On the one hand, as another way of time-frequency transfer, the concentration capability of DCT is superior to that of DFT. In this regard, [10] has shown that the DCT is close to optimal in terms of energy-compaction capabilities. Our simulation results indicate that for the same wideband communication signal, representation of signal in DCT domain is much sparser than that in DFT domain. Higher sparsity not only means lower computation complexity and shorter processing time but also means lower minimal sampling rate (compression rate) and much more accurate reconstruction. On the other hand, the DCT uses only real arithmetic, as opposed to the complex-valued DFT. This further reduces the signal-processing complexity/power consumption, especially for real-valued signal samples. As a result of the aforementioned properties, same reconstruction accuracy at a lower implementation complexity and compression rate can be achieved by DCT-CSS algorithm.

The remainder of this paper is organized as follows. In Section 2, the system model is given. Then we introduce comparisons between DCT-CSS and DFT-CSS schemes through coefficients analysis in Section 3.  Section 4 proposes the DCT-CSS scheme. Simulation results are presented in Section 5. Finally, we draw our conclusions in Section 6.

## 2. System Model

### 2.1. Signal Model

**Figure 1:**
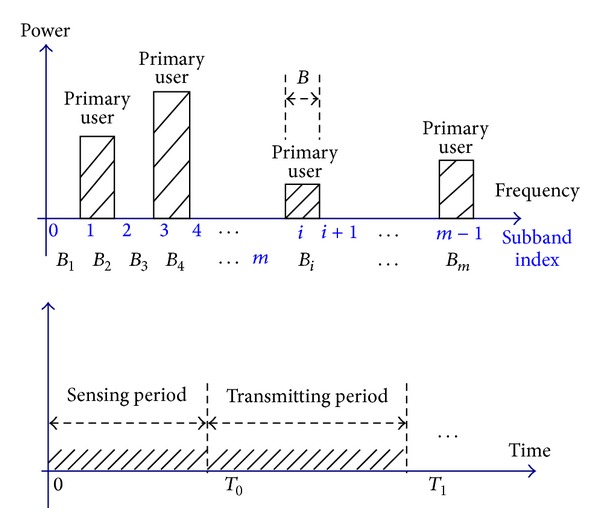
Wideband signal model.

We assume that *r*(*t*) is a band-limited signal spanning in a wide spectrum, as shown in Figure 1.

1. The wideband is divided into *m* subbands {*B*_1_, *B*_2_,…, *B*_*m*_}, and the frequency boundaries are known to the CR. The bandwidth of the spectrum bands occupied by each PU is equally *B*.
2. The signal power spectrum density (PSD) over each spectrum subband *B*_*i*_ is smooth; however, the PSD of PUs over two neighboring subbands is independent.
3. The number of active PU subbands *Q* and their locations are unknown to the CR nodes.
4. During the spectrum sensing period, all CRs keep quiet as enforced by protocols, for example, at the media access control layer.
5. In a sensing period, the locations and the number of active subbands *Q* keep unchanged but may vary for different sensing period.

### 2.2. Compressed Sampling

In practice, a signal can always be sparsely or near sparsely represented on a transform domain. For a time window as *t* ∈ [0, *τ*], *r*(*t*) have discrete form as an *N*-length signal *r*_*t*_, which can easily be described as

(1) $$\begin{array}{c} r_{t}=Fr_{f}, \end{array}$$

where the *N* × 1 vector *r*_*f*_ is the *K*-sparse frequency representation of *r*_*t*_ and *K* is the number of nonzero elements of *r*_*f*_ (*K* ≪ *N*). *F* is an *N* × *N* DFT matrix, and *ψ*_*i*_  (*i* = 1,2,…*N*) is the similarly sampled basis function.

For Nyquist theorem *N* samples are necessary to exactly reconstruct the power spectrum density. As mentioned above, the CS is able to accurately reconstruct signals only with a small portion of samples with size of *M* (*M* ≪ *N*)

(2) $$\begin{array}{c} y=\Phi r_{t} \end{array}$$

in which *y* denotes an *M*-length measurement vector, and Φ is the measurement matrix. The spectrum of *r*(*t*) can accurately be reconstructed when the measurement **y** is available. We aim at developing a spectrum sensing scheme with fewer nonadaptive measurements. Here, we use a modulated wideband converter (MWC) [11], which aims at sampling wideband sparse signal at a rate lower than that of Nyquist.

### 2.3. Reconstruction

Substituting (1) into (2), we can obtain

(3) $$\begin{array}{c} y=\Phi F^{-1}r_{f}. \end{array}$$

The reconstruction of *r*_*t*_ could be resorted to the reconstruction of *r*_*f*_

(4) $$\begin{array}{c} {\hat{r}}_{f}=\arg\underset{r_{f}}{\min}{||r_{f}||}_{0}\;\;\text{s}.\text{t}.\left(\Phi F^{-1}\right)r_{f}=y. \end{array}$$

It can be seen that (4) is a nonconvex problem. Equation (4) has a unique solution when the following holds:

(5) $$\begin{array}{c} {\hat{r}}_{f}=\arg\underset{r_{f}}{\min}{||r_{f}||}_{1}\;\;\text{s}.\text{t}.\left(\Phi F^{-1}\right)r_{f}=y. \end{array}$$

Actually, (5) is a second-order cone program. On the other hand, some variants of LASSO algorithm have been developed to deal with the noisy signals by minimizing the usual sum of squared errors:

(6) $$\begin{array}{c} {\hat{r}}_{f}=\arg\underset{r_{f}}{\min}{||r_{f}||}_{1}\;\;\text{s}.\text{t}.{||\left(\Phi F^{-1}\right)r_{f}-y||}_{2}<\varepsilon , \end{array}$$

where bounds the noise in signals. A number of convex optimization software packages have been developed to solve the LASSO problem, for example, [12].

In our work, we use (6) to solve the reconstruction problem.

## 3. Comparison between DCT and DFT

For a normal signal, it is not difficult to find a sparse representation in a certain space, where *ε* bounds the noise in signals. Actually, signals involved in CRNs have been proved sparse in the frequency domain. So, it is possible to find the unoccupied spectrum in CRNs with compressed spectrum sensing with a rate lower than Nyquist.

For the consideration of better performance of energy concentration, we present an algorithm of DCT-based compressed spectrum sensing for the wideband frequency sparse signal.

The DCT sequence is represented by

(7) V(k)=2∑n=0N−1x(n)cos[πN(n+12)k],k=0,1,…,N−1.

The DFT sequence is represented by

(8) X(k)=∑n=0N−1x(n)e−j(2πnk/N)=∑n=0N−1x(n)WNkn,k=0,1,…,N−1,

where by definition *W*_*N*_^*kn*^ = *e* ^−*j*2*π*/*N*^.

Let *s*(*n*) be a 2*N* point even symmetry extension of *x*(*n*)defined by

(9) s(n)={x(n),0≤n≤N−1x(2N−n−1),N≤n≤2N−1.

The 2*N*-point DFT of *s*(*n*) is given by

(10) $$\begin{array}{c} S\left(k\right)=\overset{2N-1}{\underset{n=0}{\sum }}s\left(n\right)W_{2N}^{kn},\;k=0,1,\ldots ,2N-1. \end{array}$$

Substituting (9) in (10) yields

(11) $$\begin{array}{c} S\left(k\right)=\overset{N-1}{\underset{n=0}{\sum }}x\left(n\right)W_{2N}^{kn}+\overset{2N-1}{\underset{n=N}{\sum }}x\left(2N-n-1\right)W_{2N}^{kn}. \end{array}$$

If we change the second index of summation using *n* = 2*N* − 1 − *m*, we recall that *W*_2*N*_^2*mN*^ = 1 for integer *m*, we factor out *W*_2*N*_^−*k*/2^, and we obtain

(12) S(k)=W2N−k/2∑n=0N−1x(n)[W2NknW2Nk/2+W2N−knW2N−k/2],k=0,1,…,2N−1.

The last expression may be written as

(13) S(k)=W2N−k/22∑n=0N−1x(n)cos[πN(n+12)k],k=0,1,…,2N−1.

Or equivalently

(14) S(k)=W2N−k/22Re[W2Nk/2∑n=0N−1x(n)W2Nkn],k=0,1,…,2N−1.

Substituting (7) in (13) yields,

(15) $$\begin{array}{c} S\left(k\right)=W_{2N}^{-k/2}V\left(k\right),\;k=0,1,\ldots ,N-1\\ \text{or}\;V\left(k\right)=W_{2N}^{k/2}S\left(k\right),\;k=0,1,\ldots ,N-1, \end{array}$$

(16) V(k)=2Re[W2Nk/2∑n=0N−1x(n)W2Nkn], k=0,1,…,N−1.

*Re*[□] implies the real part of the term enclosed.

DCT of *x*(*n*) can be computed by taking the 2*N*-point DFT of *s*(*n*), as in (9), and multiplying the result by *W*_2*N*_^*k*/2^, as in (15). Another approach is to take the 2*N*-point DFT of *x*(*n*) with *N* zeros appended to it, multiply the result by *W*_2*N*_^*k*/2^, and then take twice the real part. We note that *V*(*k*) is real and *S*(*k*) is complex.

**Figure 2:**
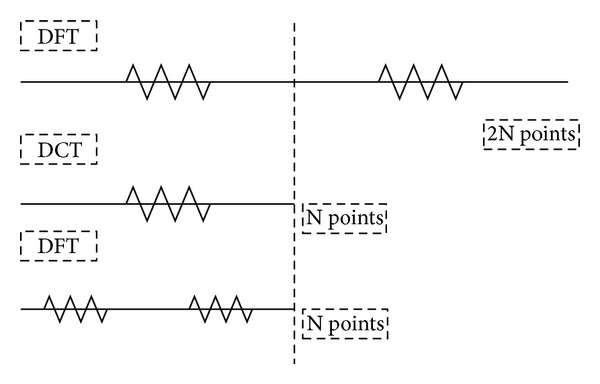
Comparison of coefficients of DCT and DFT.

As shown in Figure 2, we can easily find that the sparsity of DCT is half of the DFT.

**Figure 3:**
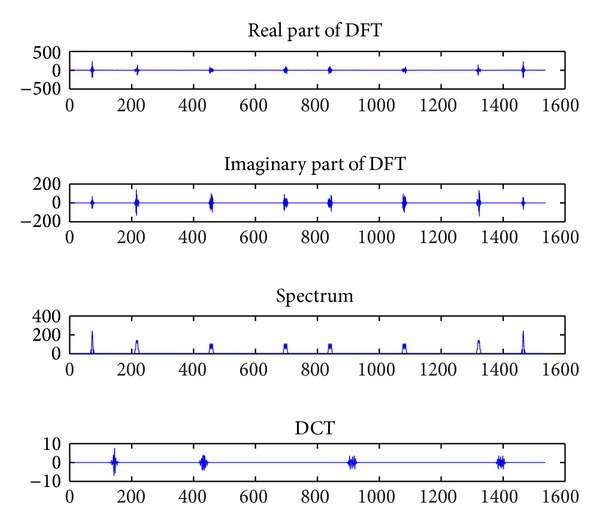
Comparison of coefficients of DCT and DFT for wideband signal.

As shown in Figure 3, we also compare the sparsity of our wideband signal between DCT and DFT. The wideband is only occupied by four channels, each of which is modulated by BPSK mode. From Figure 3, we apparently prove the validity of the conclusion we obtained above.

As discussed above, DCT has more superiorities than DFT in three aspects.

1. Transform energy compaction capability means the capability of the transform to redistribute signal energy into small number of transform coefficients. The DCT basis has better spectral compaction and energy concentration properties than DFT. That is, signal representation is much sparser in DCT domain than in DFT. This, in turn, leads to improved performance with reconstruction accuracy and can result in released computation complexity.
2. On the other hand, the DCT uses only real arithmetic, as opposed to the complex-valued DFT. Times of multiplication needed in real arithmetic account at most half of complex arithmetic. This reduces the signal-processing complexity/power consumption, especially for real-valued signal samples.
3. Both of the two points mentioned above reduced computation complexity, which in other words means less processing time. This is an important parameter in scenarios with strict time limitation, such as dynamic spectrum access.

## 4. DCT Based Compressed Spectrum Sensing

As discussed in the preceding section, the signal response is sparse in DCT domain, so the DCT-CSS problem can be solved with a three-step scheme: (1) use compressed measurements **y** to estimate the sparse sequence ${\hat{r}}_{d}$ , (2) reconstruct signal ${\hat{r}}_{t}$ according to ${\hat{r}}_{d}$ , which can be done by an inverse DCT transfer, and (3) get frequency response ${\hat{r}}_{f}$ from ${\hat{r}}_{t}$ via a fast Fourier transform (FFT).

The mathematical description of the DCT-CSS scheme is similar to the DFT-CSS scheme with Fourier matrices being replaced by the DCT matrix:

(17) $$\begin{array}{c} y=\Phi D^{-1}r_{d}, \end{array}$$

where *r*_*d*_ = *Dr*_*t*_ is the representation of *r*_*t*_ in DCT domain.

Similar to (6), we can get the estimate response ${\hat{r}}_{d}$ from

(18) $$\begin{array}{c} {\hat{r}}_{d}=\arg\underset{r_{d}}{\min}{||r_{d}||}_{1}\;\;\text{s}.\text{t}.{||\left(\Phi F^{-1}\right)r_{d}-y||}_{2}<\varepsilon . \end{array}$$

We can easily get ${\hat{r}}_{t}$ from the inverse transform ${\hat{r}}_{t}=D^{-1}{\hat{r}}_{d}$ and finally get frequency estimate

(19) $$\begin{array}{c} {\hat{r}}_{f}=\text{FFT}\left({\hat{r}}_{t}\right). \end{array}$$

## 5. Simulation

In this section, we evaluate the performance of the proposed DCT-CSS scheme. We consider that a wideband spectrum occupies 512 MHz band, which is divided into 16 subbands. We use 8 MHz BPSK modulated signal to be active signal in each band. The received signal is corrupted by additive white Gaussian noise (AWGN). On average, four subbands are occupied simultaneously, while the locations and amplitudes change for several time bursts.

We compare the normalized MSE of the estimated spectrum, which is defined as

(20) $$\begin{array}{c} \text{MSE}=E\left\{\frac{{||{\hat{r}}_{f}-r_{f}||}_{2}^{2}}{{||r_{f}||}_{2}^{2}}\right\}. \end{array}$$

**Figure 4:**
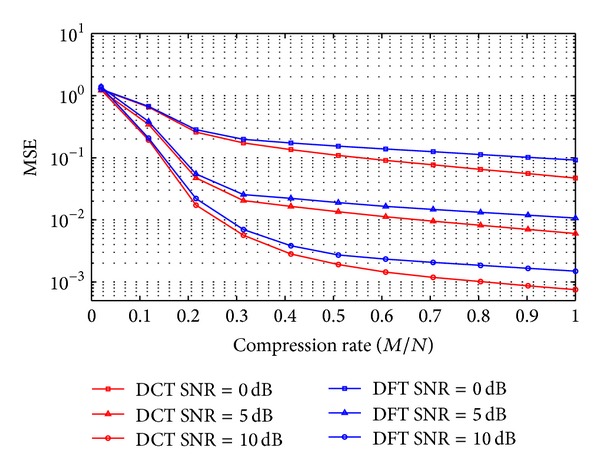
MSE performance of DCT-CSS and DFT-CSS.

From Figure 4, we can see that DCT-CSS outperforms DFT-CSS in terms of MSE of recovery as the compression rate varies from 0.05 to 1. As compression rate increases, recovery accuracy improves. On the other hand, different SNR leads to different recovery accuracy. Higher SNR means higher accuracy.

**Figure 5:**
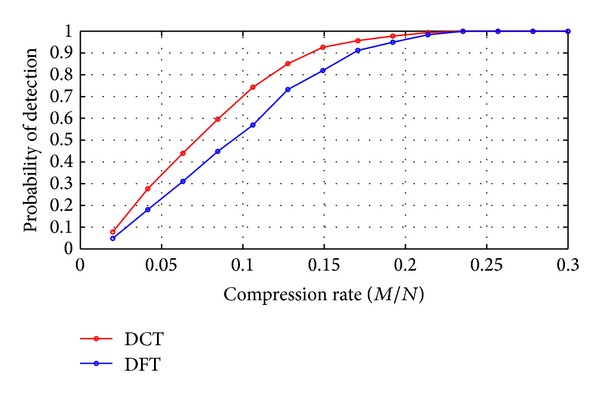
Probability of detection for DCT-CSS and DFT-CSS.

Figure 5 compares probability of detection as the compression rate varying from 0.02 to 0.3. As compression rate increases above 0.25, probability of detection of both the two schemes reaches 1 and goes to balance. However, during the region 0.02 to 0.22, DCT-CSS performs always better than DFT-CSS.

**Figure 6:**
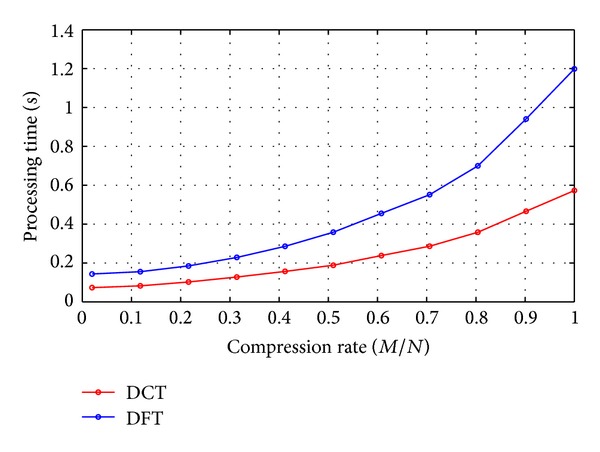
Processing time of DCT-CSS and DFT-CSS.

As shown in Figure 6, we compare the computation complexity of the two schemes. Processing time increases as the compression rate varies from 0.02 to 1. However, the processing time of DCT-CSS is approximately half of the DFT-CSS.

## 6. Conclusion

In this paper, we have proposed a novel DCT-CSS scheme for wideband spectrum sensing. Analysis has verified that wideband spectrum signal is sparser in DCT domain than in DFT. Simulation results have shown that DCT-CSS can not only improve the reconstruction accuracy and probability of detection but also save processing time. Our future work will focus on DCT-CSS scheme implemented by other reconstruction algorithms.
